# Plasma fractalkine is a sustained marker of disease severity and outcome in sepsis patients

**DOI:** 10.1186/s13054-015-1125-0

**Published:** 2015-11-25

**Authors:** Arie J. Hoogendijk, Maryse A. Wiewel, Lonneke A. van Vught, Brendon P. Scicluna, Hakima Belkasim-Bohoudi, Janneke Horn, Aeilko H. Zwinderman, Peter M. C. Klein Klouwenberg, Olaf L. Cremer, Marc J. Bonten, Marcus J. Schultz, Tom van der Poll, on behalf of the MARS consortium

**Affiliations:** Center for Experimental and Molecular Medicine, Academic Medical Center, University of Amsterdam, Meibergdreef 9, G2-130, 1105 AZ Amsterdam, The Netherlands; Department of Intensive Care, Academic Medical Center, University of Amsterdam, Amsterdam, The Netherlands; Clinical Epidemiology Biostatistics and Bioinformatics, Academic Medical Center, University of Amsterdam, Amsterdam, The Netherlands; Division of Infectious Diseases, Academic Medical Center, University of Amsterdam, Amsterdam, The Netherlands; Department of Intensive Care Medicine, University Medical Center Utrecht, Utrecht, The Netherlands; Department of Medical Microbiology, University Medical Center Utrecht, Utrecht, The Netherlands; Julius Center for Health Sciences and Primary Care, University Medical Center Utrecht, Utrecht, The Netherlands

## Abstract

**Introduction:**

Fractalkine is a chemokine implicated as a mediator in a variety of inflammatory conditions. Knowledge of fractalkine release in patients presenting with infection to the Intensive Care Unit (ICU) is highly limited. The primary objective of this study was to establish whether plasma fractalkine levels are elevated in sepsis and associate with outcome. The secondary objective was to determine whether fractalkine can assist in the diagnosis of infection upon ICU admission.

**Methods:**

Fractalkine was measured in 1103 consecutive sepsis patients (including 271 patients with community-acquired pneumonia (CAP)) upon ICU admission and at days 2 and 4 thereafter; in 73 ICU patients treated for suspected CAP in whom this diagnosis was refuted in retrospect; and in 5 healthy humans intravenously injected with endotoxin.

**Results:**

Compared to healthy volunteers, sepsis patients had strongly elevated fractalkine levels. Fractalkine levels increased with the number of organs failing, were higher in patients presenting with shock, but did not vary by site of infection. Non-survivors had sustained elevated fractalkine levels when compared to survivors. Fractalkine was equally elevated in CAP patients and patients treated for CAP but in whom the diagnosis was retrospectively refuted. Fractalkine release induced by intravenous endotoxin followed highly similar kinetics as the endothelial cell marker E-selectin.

**Conclusions:**

Plasma fractalkine is an endothelial cell derived biomarker that, while not specific for infection, correlates with disease severity in sepsis patients admitted to the ICU.

**Electronic supplementary material:**

The online version of this article (doi:10.1186/s13054-015-1125-0) contains supplementary material, which is available to authorized users.

## Introduction

Sepsis is a life-threatening condition that represents a great health burden [1] and the most frequent cause of death in non-coronary intensive care units (ICUs) in the developed world [2]. The annual incidence of severe sepsis in the USA is estimated at 300 cases per 100,000 person-years population, which accounts for 10 % of all ICU admissions [3]. The mortality of severe sepsis and septic shock lies between 25 and 50 %, with the extent and number of organs failing as the strongest predictors of an adverse outcome [2]. The clinical syndrome of sepsis is the consequence of a deregulated immune response to infection that is injurious to the host’s tissues and organs. The harmful host response during sepsis involves both disproportionate proinflammatory and immune suppressive anti-inflammatory components [1].

Fractalkine (CX_3_CL1) is a CX_3_C chemokine, which was first described to be produced by endothelial cells [4]. Subsequent research identified several additional cellular sources of fractalkine, including epithelial cells, neurons, microglial cells, osteoblasts, smooth muscle cells, dendritic cells, lymphocytes and macrophages [5, 6]. Fractalkine has a membrane-bound form and a soluble form [7]. The membrane-bound form functions as an adhesion protein, whereas soluble fractalkine acts as a chemokine [6]. The receptor for fractalkine, CX_3_CR1, is expressed by T cells, natural killer cell subsets, brain microglia, dendritic cell subsets, blood monocytes, and thrombocytes [5, 8, 9]. Fractalkine has been implicated as a mediator in several inflammatory conditions, including atherosclerosis, atopic dermatitis, airway hyper responsiveness, multiple sclerosis, and Crohn’s disease [10–12].

Recent studies have suggested that fractalkine plays a role in the pathogenesis of sepsis. Mice with polymicrobial abdominal sepsis caused by cecal ligation and puncture (CLP) have elevated fractalkine levels in their peritoneal lavage fluid and serum [13–15]. CX_3_CR1-deficient mice have enhanced mortality after CLP, suggesting that fractalkine contributes to protective immunity during sepsis [13]. Knowledge of fractalkine levels in a setting of clinical sepsis is limited to a single study that described elevated serum concentrations in 43 patients with septic shock [16]. In the present study, we analyzed sequential plasma fractalkine levels in 1,103 sepsis patients during the first 4 days of ICU admission and determined their association with the source of infection, organ failure and survival. In addition, we evaluated the possible cellular source of fractalkine by studying healthy humans injected with endotoxin. Finally, we determined whether fractalkine levels can be discriminative of infection upon ICU admission by performing analyses in 344 patients presenting with suspected community-acquired pneumonia (CAP).

## Methods

### Study design, patients and definitions

From January 2011 through July 2013 consecutive patients presenting to the mixed ICUs of two tertiary teaching hospitals (Academic Medical Center in Amsterdam and University Medical Center Utrecht) were included. Data and plasma samples were prospectively collected as part of the molecular diagnosis and risk stratification of sepsis (MARS) project, a large prospective observational study (ClinicalTrials.gov identifier NCT01905033) [17–23]. Organ failure was defined as a score of 3 or greater on the Sequential Organ Failure Assessment (SOFA) score, except for cardiovascular failure for which a score of 1 or more was used [24]. Shock was defined as the use of vasopressors (noradrenaline) for hypotension in a dose of 0.1 mcg/kg/min during at least 50 % of the ICU day. The plausibility of infection was scored post hoc based on all available evidence and classified on a 4-point scale (none, possible, probable or definite) according to Center for Disease Control and Prevention [25] and International Sepsis Forum consensus definitions [26], as described in detail previously [17]. Readmissions and patients transferred from another ICU were excluded, except for patients referred to one of the study centers on the day of admission. Daily (on admission and at 6 a.m. thereafter) left-over EDTA plasma (obtained from blood drawn for patient care) was stored within 4 hours at –80 °C. The Medical Ethical Committees of both study centers gave approval for an opt-out consent method (IRB no. 10-056C). The Municipal Personal Records Database was consulted to determine survival up to one year after ICU admission.

The primary objective of this study was to establish whether plasma fractalkine levels are elevated in sepsis and are associated with outcome. For this we analyzed all patients with sepsis diagnosed within 24 hours of admission, defined as a ‘definite’ or ‘probable’ infection [17] combined with at least one general, inflammatory, or hemodynamic, organ dysfunction, or tissue perfusion parameters derived from the 2001 International Sepsis Definitions Conference [27]. The secondary objective was to determine whether fractalkine can assist in the diagnosis of infection upon ICU admission. For this, we selected all patients with CAP from this sepsis cohort and compared these with all patients presenting with suspected CAP, for which the clinical team started therapeutic antibiotics, but in retrospect were classified as having an infection likelihood of ‘none’, as described previously [19].

### Experimental human endotoxemia

Five healthy non-smoking male volunteers (mean age 21, range 19–22 years) were intravenously administered 4 ng/kg lipopolysaccharide (LPS) (from *Escherichia coli* O113, CC-RE lot 3), kindly provided by Dr. Anthony Suffredini (National Institutes of Health, Bethesda, MD, USA). Blood was collected in EDTA tubes immediately before injection, and 0.5, 1.0, 1.5, 2.0, 3.0, 4.0, 5.0, 6.0, 8.0, 12.0, and 20.0 hours thereafter. The study was approved by the Medical Ethics Commission of the AMC, Amsterdam, The Netherlands, and written informed consent was obtained from all subjects.

### Assays

Fractalkine, soluble E-Selectin, interleukin (IL)-6, IL-8, IL-10 and TNF-α levels were measured by cytometric bead array (BD Biosciences, San Jose, CA, USA) using a FACS Calibur flow cytometer (BD Biosciences). IL-6 levels in plasma from subjects injected with LPS were determined by Luminex multiplex assay using BioPlex 200 (BioRad, Hercules, CA, USA). Normal biomarker values were acquired from EDTA plasma from 27 age- and gender-matched healthy volunteers, from whom written informed consent was obtained.

### Statistical analysis

Data are represented by box and whisker plots. Two-group comparisons were performed with the Wilcoxon rank-sum test; for multiple groups Kruskal-Wallis analysis followed by Dunn’s test was used. The primary endpoint for association with mortality was set at day 30 after ICU admission. Mixed-effects models were used to compare survivors with non-survivors over time. The log-rank test was performed to compare survival in quartiles of fractalkine levels on ICU admission. Correlation was determined using Spearman’s rho test. Multivariable logistic regression was used to establish the independent prognostic value of fractalkine after adjustment for age, Charlson comorbidity index, body mass index, admission type and severity of disease (Acute Physiology and Chronic Health Evaluation (APACHE) IV or Sequential Organ Failure Assessment (SOFA)). Variables in the model were checked for collinearity by calculating the variance inflation factor. The area under the receiver operating characteristics (ROC) curve (AUC, or the *C* statistic) and 95 % confidence intervals (CI), considering 2000 bootstrap replicates, were determined to examine the performance of fractalkine in predicting 30-day mortality. The optimal threshold was determined using the Youden index for selection of the highest sum of sensitivity and specificity. Differences in the predictive power of fractalkine and soluble E-selectin was studied by comparing the ROC AUC models by means of DeLong’s test. Prognostic analyses were complemented with the net reclassification improvement (NRI) and the integrated discrimination improvement (IDI) [28]. Calibration of the model was performed with the Hosmer and Lemeshow goodness-of-fit test. For regression analyses, biomarker values were transformed to a log scale. All analyses were performed in R (v3.1.1). *P* <0.05 was considered statistically significant.

## Results

### Patient characteristics

Characteristics of all sepsis patients are shown in Table 1. From a total of 1,103 patients, 786 (71.3 %) survived until day 30 after ICU admission, whereas 305 (27.7 %) patients did not; 12 patients were lost to follow up. The lung was the most common source of infection, followed by abdominal and urinary tract infections; survivors and non-survivors had similar sites of infection. As expected, non-survivors presented with more severe disease, as reflected by higher APACHE IV and SOFA scores, more organs failing and more shock. Thirty-day mortality occurred in 70.2 % of patients in the ICU.

**Table 1:** Clinical characteristics and outcome of sepsis patients stratified according to survival status 30 days after admission

	All patients	Survivors	Non-survivors	*P*
	n = 1103	n = 786	n = 305	
Demographics				
Age, years, mean (SD)	61.2 (14.7)	59.7 (14.7)	64.9 (13.9)	<0.0001
Gender, male, n (%)	671 (60.8)	481 (61.2)	183 (60)	0.73
Race, white, n (%)	972 (88.8)	689 (87.7)	273 (89.5)	0.45
Body mass index, kg/m^2^, mean (SD)	26 (6.2)	26.2 (6.3)	25.5 (5.8)	0.09
Admission type, medical, n (%)	817 (74.1)	562 (71.5)	247 (81)	0.001
Charlson score, median (IQR)	4 (3–6)	4 (2–6)	5 (3–6)	0.0001
Site of infection				
Pulmonary, n (%)	478 (43.3)	337 (37.1)	135 (44.3)	0.69
Abdominal, n (%)	215 (19.5)	150 (19.1)	61 (20)	0.74
Urinary, n (%)	109 (9.9)	84 (10.7)	24 (7.9)	0.69
Other, n (%)^a^	173 (15.7)	123 (15.6)	49 (16.1)	0.93
Coinfection, n (%)	128 (11.6)	92 (11.7)	36 (11.8)	1
Severity of disease in first 24 hours				
APACHE IV score, median (IQR)	80 (63–101)	74 (59–92)	98 (81–122)	<0.0001
SOFA score, median (IQR)^b^	7 (5–9)	7 (4–9)	9 (6–12)	<0.0001
Shock, n (%)	373 (33.8)	234 (29.8)	135 (44.3)	<0.001
Mechanical ventilation, n (%)	851 (77.2)	590 (75.1)	249 (81.6)	<0.05
Renal replacement therapy, n (%)	115 (10.4)	60 (7.6)	53 (17.4)	<0.001
Outcome				
ICU length of stay, days, median (IQR)	4 (2–10)	5 (2–10)	3 (2–8)	<0.01
ICU mortality, n (%)	227 (20.6)	13 (1.7)	214 (70.2)	<0.001

12 patients were lost to follow up.^a^Site of infection: “other” includes cardiovascular infection, mediastinitis and skin infection. ^b^Central nervous system not included in score. *APACHE* Acute Physiology and Chronic Health Evaluation, *SOFA* Sequential Organ Failure Assessment

### Fractalkine levels are elevated in sepsis patients during the first 4 days after ICU admission and are associated with severity of disease

**Fig. 1:**
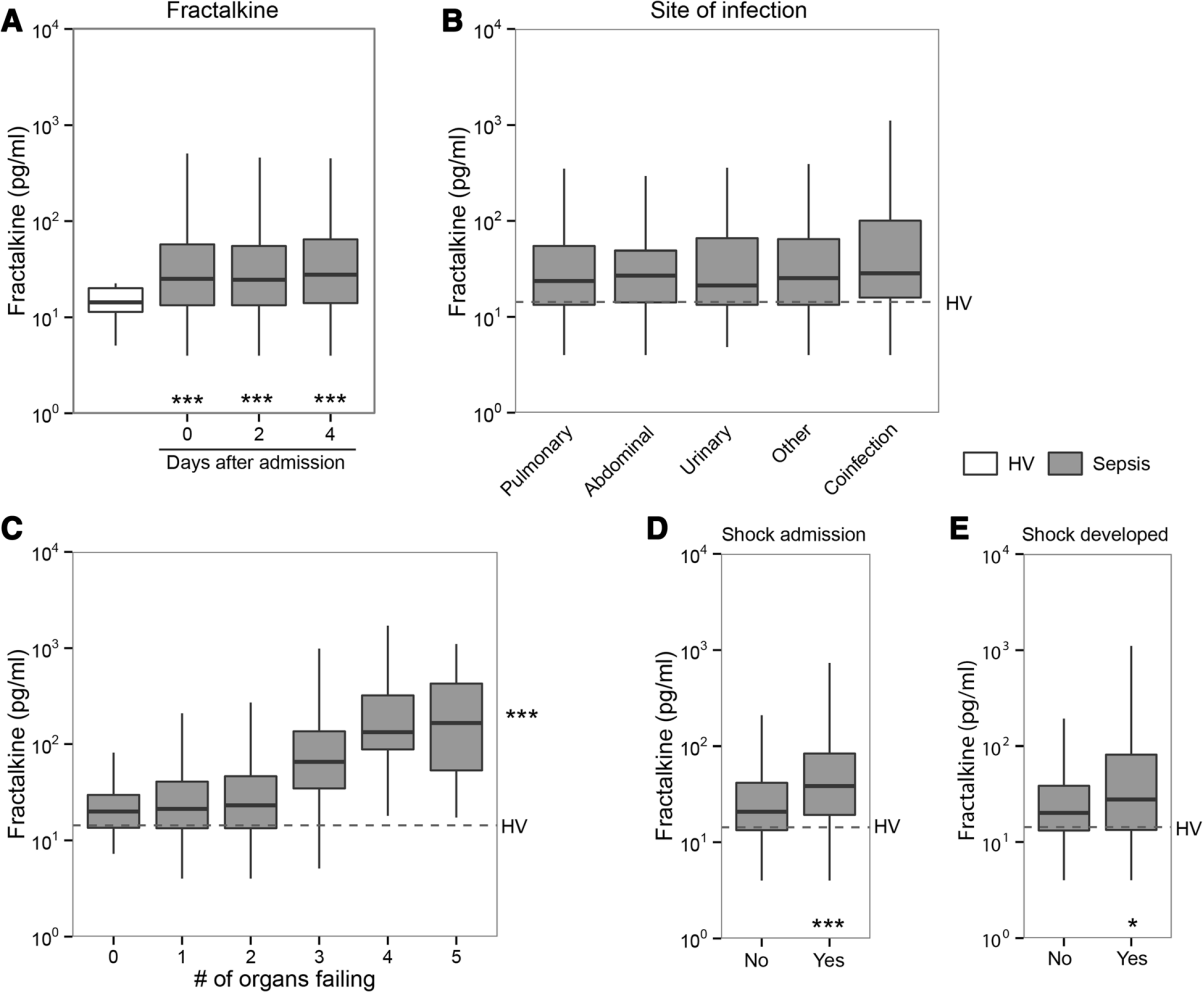
Fractalkine levels are elevated in sepsis and correlate with organ failure. Blood was drawn from patients within 24 hours of admission to the ICU (day 0) and on days 2 and 4. **a** Fractalkine levels were elevated in patients compared to healthy volunteers (*HV*) at all time points, and did not differ between days 0 and 4. **b** Fractalkine levels were not influenced by the primary source of infection. **c** Admission fractalkine levels in patients with increasing numbers of failing organs at admission. Fractalkine levels were higher in patients with shock on admission (**d**) and in patients who developed shock >24 hours after admission (**e**). *Box and whisker* diagrams depict the median and lower quartile, upper quartile, and their respective 1.5 IQR as *whiskers* (as specified by Tukey). *Gray boxes* represent sepsis; *open box* or *dotted lines* represent healthy volunteers. ***P <0.001, *P <0.05

Fractalkine levels, measured in plasma obtained within 24 hours after admission (day 0) and at days 2 and 4, were consistently elevated in patients with sepsis relative to healthy controls (Fig. 1a, *P* <0.0001 for all time points). On longitudinal analysis there was no effect of time on plasma fractalkine levels (*P* = 0.43). In contrast, in sepsis patients during the first 4 days of the ICU stay there was a time-dependent decline in plasma IL-6, IL-8 and IL-10 (Additional file 1: Figure S1, *P* <0.0001 for all analytes), which are well-known cytokine markers in sepsis [29]. Sustained elevated plasma fractalkine levels after admission for sepsis were confirmed upon separate analysis of the two contributing ICUs (data not shown). Fractalkine concentrations did not differ between sepsis patients with different sources of infection (Fig. 1b).

Admission fractalkine levels increased with increasing number of organs failing on admission (Fig. 1c, *r* = 0.42, *P* <0.001). Patients presenting with shock had significantly higher fractalkine levels than those without shock (Fig. 1d). Of the 730 patients not presenting with shock on admission, 90 (12.3 %) developed shock later during their stay on the ICU; admission fractalkine levels were higher in those septic patients who developed shock while on the ICU when compared with patients who did not (Fig. 1e).

### Fractalkine levels are higher in non-survivors

**Fig. 2:**
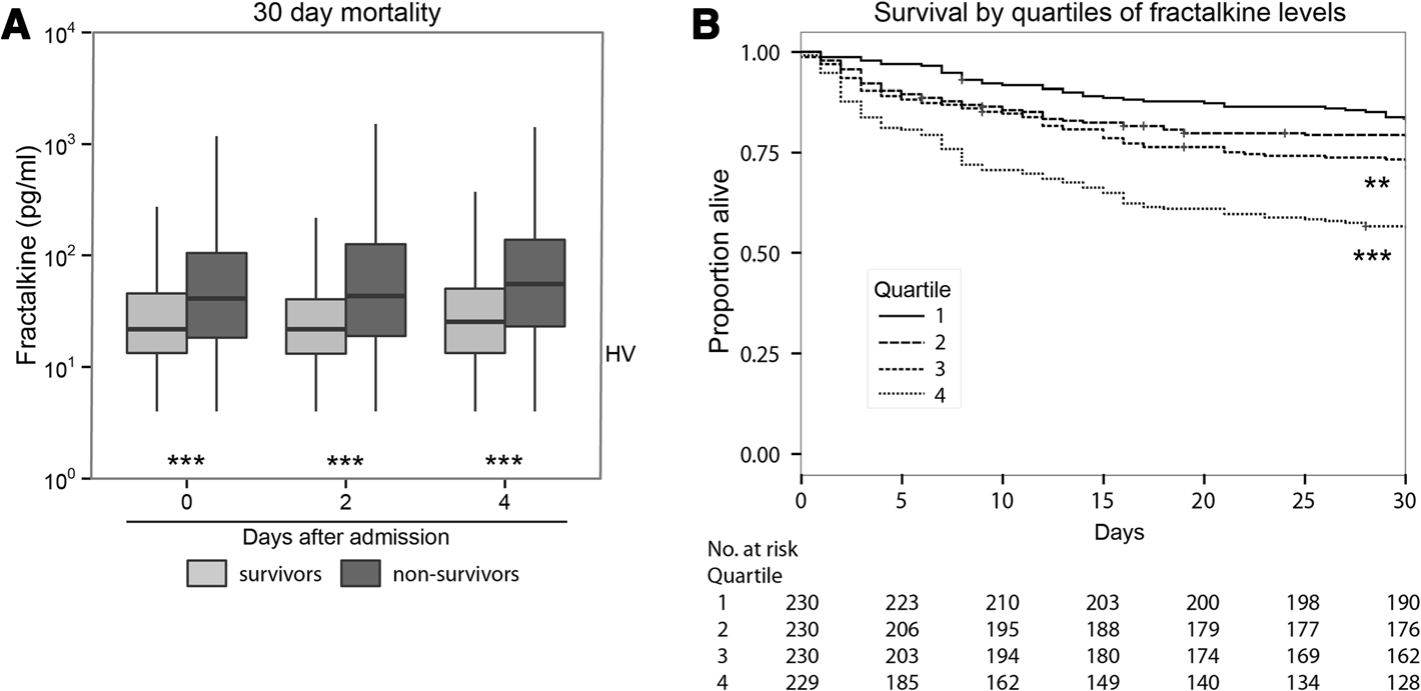
Fractalkine plasma levels are associated with 30-day mortality. Blood was drawn within 24 hours of admission to the ICU (day 0) and days 2 and 4. **a** Fractalkine levels of survivors and non-survivors at day 30, presented as *box and whiskers* as specified by Tukey. **b** Kaplan–Meier plots of survival time up to 30 days after ICU admission for quartiles (Q1 4.0–13.4 pg/ml, Q2 13.4–25.5 pg/ml, Q3 25.5–58.7 pg/ml, Q4 58.7–6329 pg/ml) of admission fractalkine levels. *Dotted lines* in box and whisker plots represent the median level in healthy volunteers (*HV*). ***P <0.001, **P <0.01

To assess the relationship between fractalkine levels and sepsis mortality, measurements were partitioned in survivors and non-survivors at day 30 after ICU admission. Fractalkine levels on admission and 2 and 4 days thereafter were significantly higher in non-survivors compared to survivors (Fig. 2a, *P* <0.001 for all time points). In a sub-analysis restricted to patients presenting with shock, fractalkine remained significantly associated with mortality (Additional file 2: Figure S2). To further investigate the association between fractalkine and 30-day mortality, admission fractalkine levels were partitioned into quartiles for which log-rank tests were performed. Quartile 3 (50–75 %, 25.5–58.7 pg/ml) and quartile 4 (75–100 %, 58.7–6329.0 pg/ml) of fractalkine levels had statistically significantly reduced survival (*P* <0.01 and *P* <0.001, respectively), compared to quartile 1 (Fig. 2b). Admission fractalkine levels remained significantly associated with 30-day mortality after adjustment for age, Charlson comorbidity index, body mass index (BMI), admission type and APACHE IV scores in a logistic regression model (odds ratio (OR), 1.37 (95 % CI, 1.19–1.58) for each log increase in fractalkine, *P* <0.0001). Similarly, when fractalkine levels were included in a logistic regression analysis together with SOFA scores, the association with 30-day mortality persisted (OR, 1.25, 95 % CI, 1.09–1.44, for each log increase in fractalkine, *P* = 0.001). Moreover, plasma levels on days 2 and 4 remained independently associated with 30-day mortality after adjustment for SOFA scores for those particular days (OR 1.40, 95 % CI 1.20–1.63, *P* = 0.001 and OR 1.31, 95 % CI 1.08–1.59, *P* = 0.006, respectively). The ROC AUC of fractalkine was 0.65 (95 % CI 0.61–0.69). The Youden index determined a fractalkine level of 38.2 pg/ml to be the optimal cutoff. At this cutoff, fractalkine had 54 % sensitivity and 71 % specificity for predicting mortality at 30 days after ICU admission. Fractalkine in addition to the APACHE IV score, improved classification of patients who were deceased at 30 days after admission and those who were not; the NRI was 0.27 (95 % CI 0.12–0.41, *P* = 0.0003) and the IDI was 0.02 (95 % CI 0.006–0.02, *P* = 0.002).

The association between elevated fractalkine concentrations and mortality persisted when outcomes were 7-day, 90-day or 1-year mortality (Additional file 3: Figure S3). Together these data suggest that higher fractalkine levels are associated with short-term mortality, and that this association drives differences in long-term mortality.

### Comparison of fractalkine release with that of soluble E-selectin

**Fig. 3:**
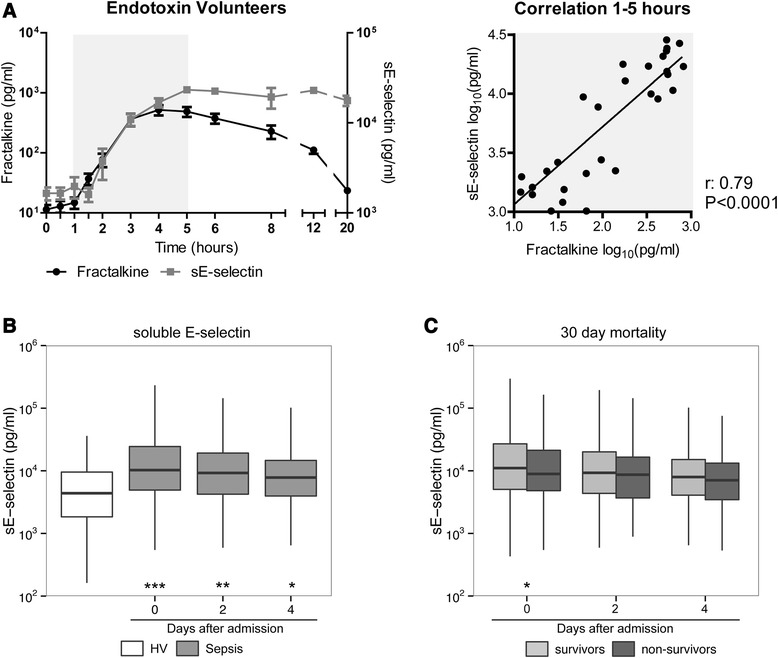
Soluble E-selectin correlates with fractalkine release after intravenous injection of endotoxin in healthy humans in vivo but is not associated with increased mortality in sepsis patients. **a** Fractalkine release after intravenous injection of endotoxin (lipopolysaccharide, 4 ng/kg body weight) into five healthy subjects compared with E-selectin release. *Right panel* shows correlation between fractalkine levels and E-selectin measured during the first 5 hours after endotoxin administration (shaded gray). Data are expressed as mean ± standard error of the mean. **b** Soluble E-selectin levels were elevated in patients compared to healthy volunteers (*HV*) at all time points. **c** Soluble E-selectin levels of survivors and non-survivors at day 30, presented as box and whiskers as specified by Tukey. ***P <0.001, **P <0.01, *P <0.05

Several studies have identified endothelial cells as a major source of fractalkine [4, 30, 31]. Considering that it is difficult to assess whether the endothelium produces fractalkine during human sepsis in vivo, we compared the kinetics of fractalkine release into the circulation relative to that of the established specific endothelial cell activation marker E-selectin [32, 33] in a controlled human setting of systemic inflammation induced by bolus intravenous injection of LPS. Intravenous LPS induced a rise in fractalkine levels with similar kinetics to the increase in soluble E-selectin (Fig. 3a, *P* <0.0001). The initial increase in soluble E-selectin coincided with the rise in fractalkine levels, and fractalkine and soluble E-selectin levels measured during the first 5 hours after LPS injection had strong positive correlation (Fig. 3a; *r* = 0.79, *P* <0.0001). The release of IL-6 occurred faster and was more transient, and IL-6 levels did not correlate with fractalkine levels (Additional file 4: Figure S4). We also measured soluble E-selectin levels in the patient samples used in Figs. 1 and 2. While soluble E-selectin levels were clearly elevated in patients with sepsis when compared with healthy controls (Fig. 3b, *P* <0.001), confirming previous reports [34, 35], soluble E-selectin was not associated with increased mortality at day 30 (Fig. 3c); admission soluble E-selectin was, in contrast with fractalkine, lower in non-survivors (*P* = 0.02). The ROC AUC of soluble E-selectin for 30-day mortality was 0.55 (95 % CI 0.51–0.59). Fractalkine outperformed soluble E-selectin in predicting 30-day mortality (*P* = 0.001).

### Elevated plasma fractalkine levels are not specific for infection

**Fig. 4:**
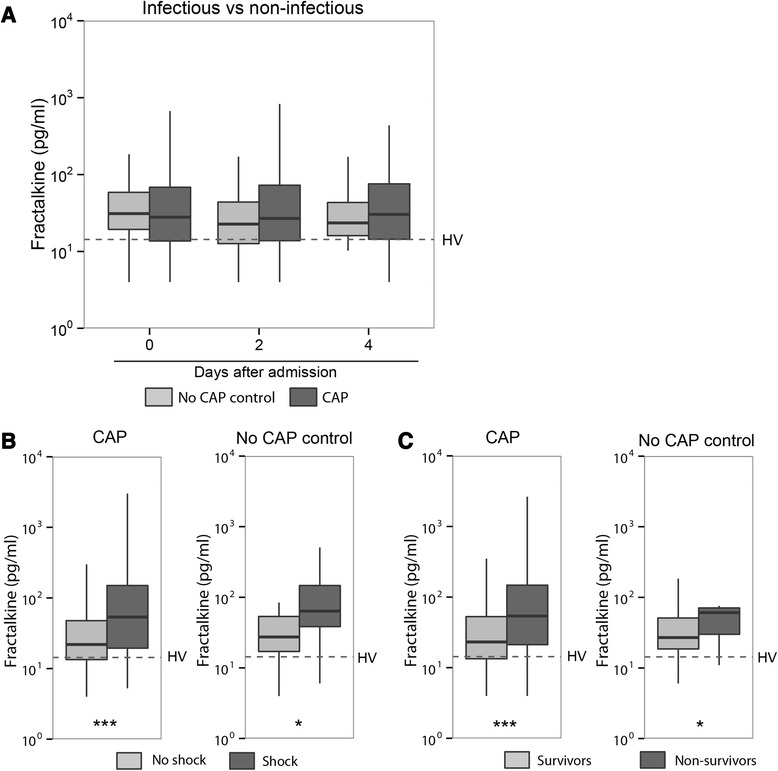
Fractalkine levels in patients with community-acquired pneumonia (*CAP*) and critically ill patients with no CAP. Blood was drawn from patients within 24 hours of admission to the ICU (day 0) and at days 2 and 4. **a** Fractalkine plasma levels in patients with suspected CAP upon ICU admission and classified in retrospect as having CAP or no CAP. Differences between groups were not significant. **b** Fractalkine levels were higher in patients with shock on admission (**b**) and in non-survivors (at day 30) (**c**) in both CAP and no-CAP patients. *Box and whisker* diagrams depict the median and lower quartile, upper quartile, and their respective 1.5 IQR as *whiskers* (as specified by Tukey). *Dotted lines* represent the median level in healthy volunteers (*HV*). ***P <0.001, *P <0.05

Considerable research has focused on discriminating infectious from non-infectious sources of critical illness. To determine whether fractalkine levels can provide diagnostic value we analyzed fractalkine levels in 344 patients consecutively admitted to the ICU with suspected CAP. Of these, 271 patients were classified as having CAP (these were also part of the sepsis cohort described above), whereas in 73 patients the CAP diagnosis was retrospectively refuted (no-CAP controls) [17]. Clinical characteristics of patients with and without CAP are depicted in Table 2. these patients were largely similar in demographics, comorbidities, severity of disease and outcome. Patients with CAP more often had shock and had longer lengths of stay in the ICU. The ICU mortality and 30-day mortality did not differ between groups. Fractalkine levels were similar in patients with and without CAP on admission and at 2 and 4 days thereafter (Fig. 4a). Fractalkine levels were higher in patients with CAP and without CAP who presented with shock than in those who did not (Fig. 4b); similarly, fractalkine levels were higher in patients with and without CAP who did not survive until day 30, when compared with those who did (Fig. 4c).

**Table 2:** Clinical characteristics and outcome of patients with community-acquired pneumonia (CAP) and no-CAP controls

	CAP	No CAP	*P*
	n = 271	n = 73	
Demographics			
Age, years, mean (SD)	59.7 (16.7)	58.8 (17.2)	0.70
Gender, male, n (%)	155 (57.2)	40 (54.8)	0.79
Race, white, n (%)	236 (87.1)	57 (78.1)	0.04
Body mass index, kg/m^2^, mean (SD)	24.9 (6.0)	26.4 (9.4)	0.21
Charlson score, median (IQR)	4 (2–6)	3 (2–5)	0.07
Severity of disease in first 24 hours			
APACHE IV score, median (IQR)	79 (62–101)	68 (52–99)	0.07
SOFA score, median (IQR)^a^	7 (4–9)	6 (3–7)	0.01
Shock, n (%)	71 (26.2)	10 (13.7)	0.03
Mechanical ventilation, n (%)	203 (74.9)	57 (78.1)	0.66
Renal replacement therapy, n (%)	19 (7)	4 (5.5)	0.78
Outcome			
ICU length of stay, days, median (IQR)	5 (2–11)	2 (1–4)	<0.0001
ICU mortality, n (%)	53 (19.6)	9 (12.3)	0.18
30-day mortality, n (%)	73 (26.9)	16 (21.9)	0.37
90-day mortality, n (%)	92 (33.9)	19 (26)	0.16
1-year mortality, n (%)	121 (44.6)	30 (41.1)	0.50

All patients were treated for suspected CAP upon ICU admission and classified in retrospect as having or not having CAP, as described in “Methods”. ^a^Central nervous system not included in score. *APACHE* Acute Physiology and Chronic Health Evaluation, *SOFA* Sequential Organ Failure Assessment

## Discussion

Fractalkine is the only member of the CX3C class of chemokines, and thus far, primarily studied as a potential mediator of chronic inflammatory conditions. We performed a detailed analysis of plasma fractalkine concentrations during the first 4 days after ICU admission in almost 1,200 acutely ill patients to show that elevated plasma fractalkine, while not specific for the presence of infection, is a sustained marker of disease severity and an adverse outcome in sepsis patients admitted to the ICU.

Fractalkine may not be merely a marker of disease severity in sepsis. Animal studies have suggested an important functional role for fractalkine in the pathogenesis of sepsis. In accordance with our human data, abdominal sepsis induced by CLP in mice was associated with elevated plasma fractalkine levels [14, 15]. Elimination of the biological activity of fractalkine, achieved by studying mice deficient for its receptor CX3CR1, has been shown to exaggerate organ damage and increase lethality [13]. During murine abdominal sepsis, fractalkine mRNA levels are elevated in the heart, lung, kidney, and liver, indicating that many organs contribute to elevated plasma concentrations [15].

Although fractalkine can be produced by a variety of cell types, arterial and capillary endothelial cells have been identified as a major source during endotoxemia in rats [31]. In accordance, several studies have reported the capacity of endothelial cells to produce fractalkine [4, 30, 31]. As endothelial cells are not easily accessible for studying production of inflammatory mediators in patients with sepsis, we used the model of human endotoxemia to obtain indirect evidence for the endothelium as an important source of fractalkine, comparing the release of fractalkine with that of E-selectin. We regarded E-selectin a relevant biomarker in this context, considering that it is endothelial-cell-specific [36], and that increased endothelial cell expression of fractalkine and E-selectin by inflammatory stimuli is mediated by similar mechanisms involving activation of NF-κB [30, 37]; moreover, the release of both fractalkine and E-selectin from endothelial cells is regulated by members of the family of disintegrins and metalloproteinases [38, 39]. We indeed found remarkably similar kinetics for the appearance of fractalkine and E-selectin in the circulation after intravenous injection of LPS, and fractalkine levels were positively and significantly correlated with E-selectin levels. Together these data suggest endothelial cells are a major source of fractalkine in sepsis. Blood leukocytes are an unlikely source of fractalkine in sepsis, as whole blood leukocytes did not release fractalkine upon stimulation with Toll-like receptor ligands relevant for common sepsis pathogens (data not shown). In accordance, fractalkine mRNA levels were undetectable in blood leukocytes from a subgroup of 440 sepsis patients in whom the genome-wide transcriptome was assessed (data not shown).

A substantial number of biomarkers have been proposed for the diagnostic stratification of infectious and non-infectious ICU patients [29], most notably procalcitonin [40]. To the best of our knowledge, published data on fractalkine levels in an ICU setting is limited to a single study reporting fractalkine concentrations in 43 patients with septic shock [16]. Considering that circulating fractalkine concentrations are elevated in a variety of chronic non-infectious inflammatory conditions [41–45], we argued that elevated fractalkine levels are likely not specific for the presence of infection upon ICU admission. To address this, we measured fractalkine levels in critically ill patients suspected of having CAP upon ICU admission, who in retrospect were classified as having an infection likelihood of ‘definite’ or ‘probable’ (CAP patients), or ‘none’ (no-CAP patients). The likelihood of the CAP diagnosis was classified by dedicated research physicians by post hoc review of all available clinical, radiological, and microbiological evidence, and using strict diagnostic criteria [17]; the interobserver agreement for the diagnosis and likelihood of CAP was excellent [17]. By using this additional ICU cohort we clearly show that fractalkine cannot be considered a marker for infection in critically ill patients. Also elevated fractalkine levels were associated with increased disease severity and an adverse outcome in patients without CAP.

How could fractalkine levels be of use at the bedside? Many different biomarkers for diagnosis of infection and ICU prognosis have been described in the literature, and none of these are currently used in clinical practice [29]. The challenge for future clinical implementation is to define combinations of biomarkers concurrently measured at the bedside that together with clinical readouts provide useful information for detection of infection and risk stratification [46]. The current study suggests that plasma fractalkine levels could be part of a test aiding in risk stratification during the first days after ICU admission.

## Conclusions

This large observational study suggests that plasma fractalkine, while not specific for infection, is a sustained biomarker of disease severity and outcome in sepsis patients admitted to the ICU, irrespective of the primary source of infection and is likely derived from the activated vascular endothelium.

## Key messages

- Plasma fractalkine is an endothelial-cell-derived biomarker
- Fractalkine levels are elevated in sepsis patients during the first 4 days after ICU admission
- Fractalkine levels are strongly associated with severity of disease and adverse outcome
- Elevated plasma fractalkine levels are not specific for infection in the critically ill

## Additional files

Additional file 1: Figure S1. Elevated IL-6, IL-8 and IL-10 levels in sepsis patients. Blood was drawn from patients within 24 hours of admission to the ICU (day 0) and at days 2 and 4. IL-6 (**a**), IL-8 (**b**) and IL-10 (**c**) levels were measured in plasma. *Box and whisker* diagrams depict median and lower quartile, upper quartile, and their respective 1.5 IQR as *whiskers* (as specified by Tukey). *Gray boxes*, sepsis patients (n = 1,103), *open boxes*, healthy volunteers (*HV*, n = 27). ****P* <0.001 for sepsis patients compared to HV and for decline over time. (DOC 151 kb) Additional file 2: Figure S2. Fractalkine plasma levels are associated with mortality in patients with shock within 24 hours of admission. Patient blood was drawn within 24 hours of admission to the ICU (day 0) and at days 2 and 4. Fractalkine levels of 30-day survivors and non-survivors in a subgroup of patients admitted with shock. *Box and whisker* diagrams depict median and lower quartile, upper quartile, and their respective 1.5 IQR as *whiskers* (as specified by Tukey). *Dotted lines* in boxes, median level in healthy volunteers (*HV*). ***P* <0.01, **P* <0.05. (DOC 83 kb) Additional file 3: Figure S3. Elevated fractalkine plasma levels are associated with short- and long-term mortality. Patient blood was drawn within 24 hours of admission to the ICU (day 0) and at days 2 and 4. Fractalkine levels are shown for survivors and non-survivors at day 7 (**a**), day 90 (**b**) and one year (**c**) after ICU admission. *Box and whisker* diagrams depict median and lower quartile, upper quartile, and their respective 1.5 IQR as *whiskers* (as specified by Tukey). *Dotted lines in boxes*, median level in healthy volunteers. ****P* <0.001. (DOC 81 kb) Additional file 4: Figure S4. Fractalkine compared to IL-6 release after intravenous injection of endotoxin to healthy humans in vivo. Fractalkine release after intravenous injection of endotoxin (lipopolysaccharide, 4 ng/kg body weight) into five healthy subjects compared with IL-6 release. *Right panel*, correlation between fractalkine levels and IL-6 measured during the first 5 hours after endotoxin administration (*gray*). Data are expressed as mean ± standard error of the mean. (DOC 135 kb)

## Abbreviations

APACHE: Acute Physiology and Chronic Health Evaluation
AUC: area under the curve
BMI: body mass index
CAP: community-acquired pneumonia
CI: confidence interval
CLP: cecal ligation and puncture
ICU: intensive care unit
IDI: integrated discrimination improvement
IL: interleukin
LPS: lipopolysaccharide
NRI: net reclassification improvement
OR: odds ratio
ROC: receiver operating characteristic
SOFA: Sequential Organ Failure Assessment
TNF: tumor necrosis factor

## References

[1] Angus DC, van der Poll T (2013). Severe sepsis and septic shock. N Engl J Med..

[2] Mayr FB, Yende S, Angus DC (2014). Epidemiology of severe sepsis. Virulence..

[3] Angus DC, Linde-Zwirble WT, Lidicker J, Clermont G, Carcillo J, Pinsky MR (2001). Epidemiology of severe sepsis in the United States: analysis of incidence, outcome, and associated costs of care. Crit Care Med..

[4] Bazan JF, Bacon KB, Hardiman G, Wang W, Soo K, Rossi D (1997). A new class of membrane-bound chemokine with a CX3C motif. Nature..

[5] Landsman L, Bar-On L, Zernecke A, Kim KW, Krauthgamer R, Shagdarsuren E (2009). CX3CR1 is required for monocyte homeostasis and atherogenesis by promoting cell survival. Blood..

[6] Corcione A, Ferretti E, Pistoia V (2012). CX3CL1/fractalkine is a novel regulator of normal and malignant human B cell function. J Leukoc Biol..

[7] Garton KJ, Gough PJ, Blobel CP, Murphy G, Greaves DR, Dempsey PJ (2001). Tumor necrosis factor-alpha-converting enzyme (ADAM17) mediates the cleavage and shedding of fractalkine (CX3CL1). J Biol Chem..

[8] Miksa M, Amin D, Wu R, Ravikumar TS, Wang P (2007). Fractalkine-induced MFG-E8 leads to enhanced apoptotic cell clearance by macrophages. Mol Med..

[9] Schulz C, Schafer A, Stolla M, Kerstan S, Lorenz M, von Bruhl ML (2007). Chemokine fractalkine mediates leukocyte recruitment to inflammatory endothelial cells in flowing whole blood: a critical role for P-selectin expressed on activated platelets. Circulation..

[10] Ferretti E, Pistoia V, Corcione A (2014). Role of fractalkine/CX3CL1 and its receptor in the pathogenesis of inflammatory and malignant diseases with emphasis on B cell malignancies. Mediators Inflamm..

[11] Jones BA, Beamer M, Ahmed S (2010). Fractalkine/CX3CL1: a potential new target for inflammatory diseases. Mol Interv..

[12] D’Haese JG, Friess H, Ceyhan GO (2012). Therapeutic potential of the chemokine-receptor duo fractalkine/CX3CR1: an update. Expert Opin Ther Targets..

[13] Ishida Y, Hayashi T, Goto T, Kimura A, Akimoto S, Mukaida N (2008). Essential involvement of CX3CR1-mediated signals in the bactericidal host defense during septic peritonitis. J Immunol..

[14] He M, Moochhala SM, Adhikari S, Bhatia M (2009). Administration of exogenous fractalkine, a CX3C chemokine, is capable of modulating inflammatory response in cecal ligation and puncture-induced sepsis. Shock..

[15] Raspé C, Höcherl K, Rath S, Sauvant C, Bucher M (2013). NF-κB-mediated inverse regulation of fractalkine and CX3CR1 during CLP-induced sepsis. Cytokine..

[16] Pachot A, Cazalis MA, Venet F, Turrel F, Faudot C, Voirin N (2008). Decreased expression of the fractalkine receptor CX3CR1 on circulating monocytes as new feature of sepsis-induced immunosuppression. J Immunol..

[17] Klein Klouwenberg PM, Ong DS, Bos LD, de Beer FM, van Hooijdonk RT, Huson MA (2013). Interobserver agreement of Centers for Disease Control and Prevention criteria for classifying infections in critically ill patients. Crit Care Med..

[18] Klein Klouwenberg PM, van Mourik MS, Ong DS, Horn J, Schultz MJ, Cremer OL (2014). Electronic implementation of a novel surveillance paradigm for ventilator-associated events. Feasibility and validation. Am J Respir Crit Care Med.

[19] Scicluna BP, Klein Klouwenberg PM, van Vught LA, Wiewel MA, Ong DS, Zwinderman AH (2015). A molecular biomarker to diagnose community-acquired pneumonia on intensive care unit admission. Am J Respir Crit Care Med..

[20] Bos LD, Cremer OL, Ong DS, Caser EB, Barbas CS, Villar J (2015). External validation confirms the legitimacy of a new clinical classification of ARDS for predicting outcome. Intensive Care Med.

[21] Ong DS, Bonten MJ, Safdari K, Spitoni C, Frencken JF, Witteveen E (2015). Epidemiology, management, and risk-adjusted mortality of ICU-acquired enterococcal bacteremia. Clin Infect Dis..

[22] Ong DS, Klein Klouwenberg PM, Verduyn Lunel FM, Spitoni C, Frencken JF, Dekker HA (2015). Cytomegalovirus seroprevalence as a risk factor for poor outcome in acute respiratory distress syndrome. Crit Care Med..

[23] Geboers DG, de Beer FM, Tuip-de Boer AM, van der Poll T, Horn J, Cremer OL (2015). Plasma suPAR as a prognostic biological marker for ICU mortality in ARDS patients. Intensive Care Med..

[24] Kaukonen KM, Bailey M, Suzuki S, Pilcher D, Bellomo R (2014). Mortality related to severe sepsis and septic shock among critically ill patients in Australia and New Zealand, 2000-2012. JAMA..

[25] Garner JS, Jarvis WR, Emori TG, Horan TC, Hughes JM (1988). CDC definitions for nosocomial infections, 1988. Am J Infect Control..

[26] Calandra T, Cohen J (2005). The International Sepsis Forum Consensus Conference on Definitions of Infection in the Intensive Care Unit. Crit Care Med..

[27] Levy MM, Fink MP, Marshall JC, Abraham E, Angus D, Cook D (2003). 2001 SCCM/ESICM/ACCP/ATS/SIS International Sepsis Definitions Conference. Crit Care Med..

[28] Pencina MJ, D’Agostino RB, D’Agostino RB, Vasan RS (2008). Evaluating the added predictive ability of a new marker: from area under the ROC curve to reclassification and beyond. Stat Med..

[29] Pierrakos C, Vincent JL (2010). Sepsis biomarkers: a review. Crit Care..

[30] Garcia GE, Xia Y, Chen S, Wang Y, Ye RD, Harrison JK (2000). NF-kappaB-dependent fractalkine induction in rat aortic endothelial cells stimulated by IL-1beta, TNF-alpha, and LPS. J Leukoc Biol..

[31] Sung MJ, Kim W, Ahn SY, Cho CH, Koh GY, Moon SO (2005). Protective effect of alpha-lipoic acid in lipopolysaccharide-induced endothelial fractalkine expression. Circ Res..

[32] Page AV, Liles WC (2013). Biomarkers of endothelial activation/dysfunction in infectious diseases. Virulence..

[33] Opal SM, van der Poll T (2015). Endothelial barrier dysfunction in septic shock. J Intern Med..

[34] Newman W, Beall LD, Carson CW, Hunder GG, Graben N, Randhawa ZI (1993). Soluble E-selectin is found in supernatants of activated endothelial cells and is elevated in the serum of patients with septic shock. J Immunol..

[35] Cummings CJ, Sessler CN, Beall LD, Fisher BJ, Best AM, Fowler AA (1997). Soluble E-selectin levels in sepsis and critical illness. Correlation with infection and hemodynamic dysfunction. Am J Respir Crit Care Med.

[36] Telen MJ (2014). Cellular adhesion and the endothelium: E-selectin, L-selectin, and pan-selectin inhibitors. Hematol Oncol Clin North Am..

[37] Collins T, Read MA, Neish AS, Whitley MZ, Thanos D, Maniatis T (1995). Transcriptional regulation of endothelial cell adhesion molecules: NF-kappa B and cytokine-inducible enhancers. FASEB J..

[38] Garton KJ, Gough PJ, Raines EW (2006). Emerging roles for ectodomain shedding in the regulation of inflammatory responses. J Leukoc Biol..

[39] Dreymueller D, Pruessmeyer J, Groth E, Ludwig A (2012). The role of ADAM-mediated shedding in vascular biology. Eur J Cell Biol..

[40] Wacker C, Prkno A, Brunkhorst FM, Schlattmann P (2013). Procalcitonin as a diagnostic marker for sepsis: a systematic review and meta-analysis. Lancet Infect Dis..

[41] Flierl U, Schafer A (2012). Fractalkine – a local inflammatory marker aggravating platelet activation at the vulnerable plaque. Thromb Haemost..

[42] Stolla M, Pelisek J, von Bruhl ML, Schafer A, Barocke V, Heider P (2012). Fractalkine is expressed in early and advanced atherosclerotic lesions and supports monocyte recruitment via CX3CR1. PLoS One..

[43] Staumont-Salle D, Fleury S, Lazzari A, Molendi-Coste O, Hornez N, Lavogiez C (2014). CX(3)CL1 (fractalkine) and its receptor CX(3)CR1 regulate atopic dermatitis by controlling effector T cell retention in inflamed skin. J Exp Med..

[44] Ridderstad Wollberg A, Ericsson-Dahlstrand A, Jureus A, Ekerot P, Simon S, Nilsson M (2014). Pharmacological inhibition of the chemokine receptor CX3CR1 attenuates disease in a chronic-relapsing rat model for multiple sclerosis. Proc Natl Acad Sci USA.

[45] Huo LW, Ye YL, Wang GW, Ye YG (2015). Fractalkine (CX3CL1): A biomarker reflecting symptomatic severity in patients with knee osteoarthritis. J Investig Med..

[46] Casserly B, Read R, Levy MM (2011). Multimarker panels in sepsis. Crit Care Clin..

