# Supplementary figures and images for: Plasma fractalkine is a sustained marker of disease severity and outcome in sepsis patients
Source: Crit Care. 2015 Nov 25;19:412. doi: 10.1186/s13054-015-1125-0 (PMC4658804; doi:10.1186/s13054-015-1125-0)

**Figure S1**

**
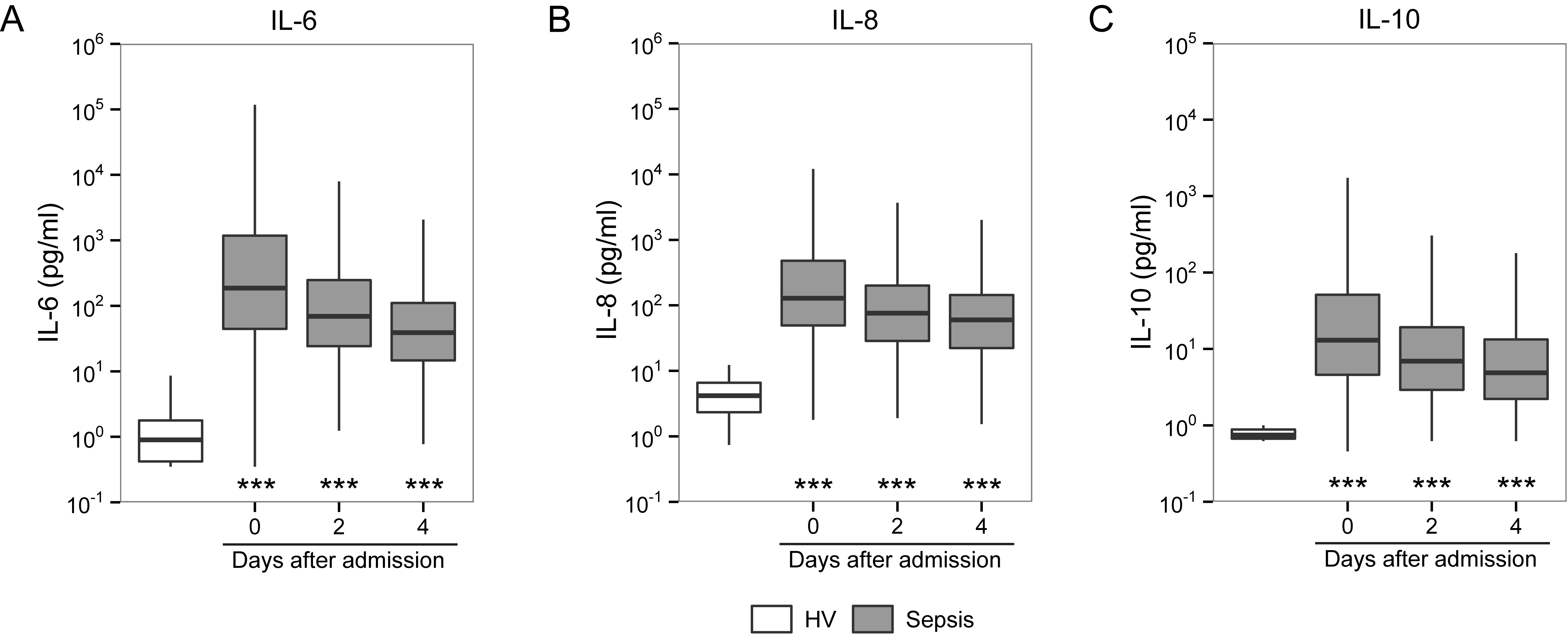
**

Supplement: Additional file 1: Figure S1. — Elevated IL-6, IL-8 and IL-10 levels in sepsis patients. Blood was drawn from patients within 24 hours of admission to the ICU (day 0) and at days 2 and 4. IL-6 (a), IL-8 (b) and IL-10 (c) levels were measured in plasma. Box and whisker diagrams depict median and lower quartile, upper quartile, and their respective 1.5 IQR as whiskers (as specified by Tukey). Gray boxes, sepsis patients (n = 1,103), open boxes, healthy volunteers (HV, n = 27). ***P <0.001 for sepsis patients compared to HV and for decline over time. (DOC 151 kb) [file 13054_2015_1125_MOESM1_ESM.doc]

**Figure S2**

**
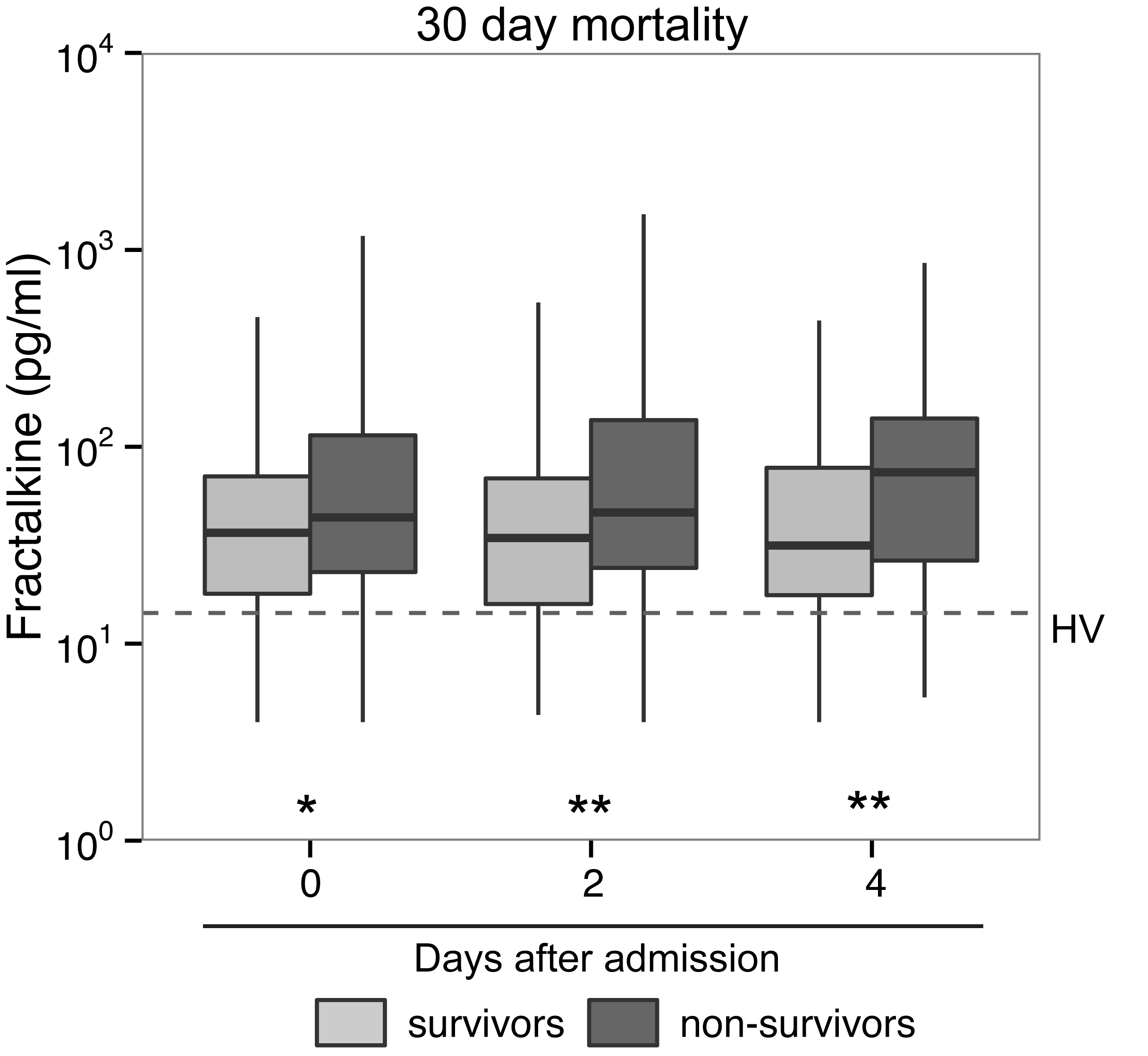
**

Supplement: Additional file 2: Figure S2. — Fractalkine plasma levels are associated with mortality in patients with shock within 24 hours of admission. Patient blood was drawn within 24 hours of admission to the ICU (day 0) and at days 2 and 4. Fractalkine levels of 30-day survivors and non-survivors in a subgroup of patients admitted with shock. Box and whisker diagrams depict median and lower quartile, upper quartile, and their respective 1.5 IQR as whiskers (as specified by Tukey). Dotted lines in boxes, median level in healthy volunteers (HV). **P <0.01, *P <0.05. (DOC 83 kb) [file 13054_2015_1125_MOESM2_ESM.doc]

**Figure S3**

**
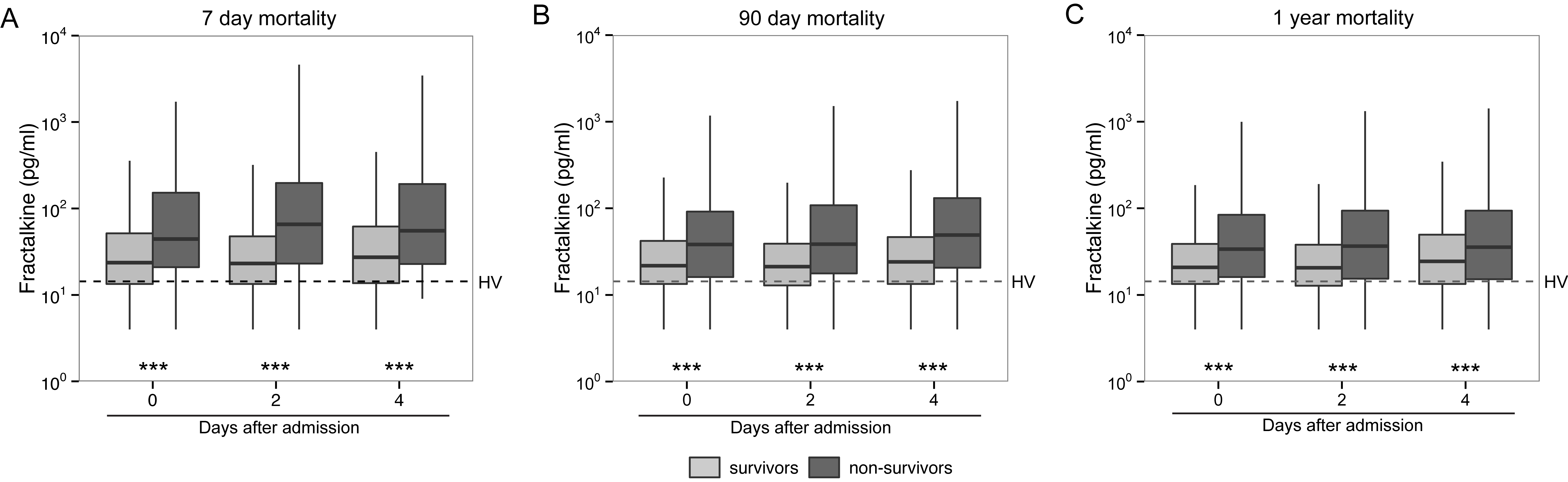
**

Supplement: Additional file 3: Figure S3. — Elevated fractalkine plasma levels are associated with short- and long-term mortality. Patient blood was drawn within 24 hours of admission to the ICU (day 0) and at days 2 and 4. Fractalkine levels are shown for survivors and non-survivors at day 7 (a), day 90 (b) and one year (c) after ICU admission. Box and whisker diagrams depict median and lower quartile, upper quartile, and their respective 1.5 IQR as whiskers (as specified by Tukey). Dotted lines in boxes, median level in healthy volunteers. ***P <0.001. (DOC 81 kb) [file 13054_2015_1125_MOESM3_ESM.doc]

**Figure S4**

**
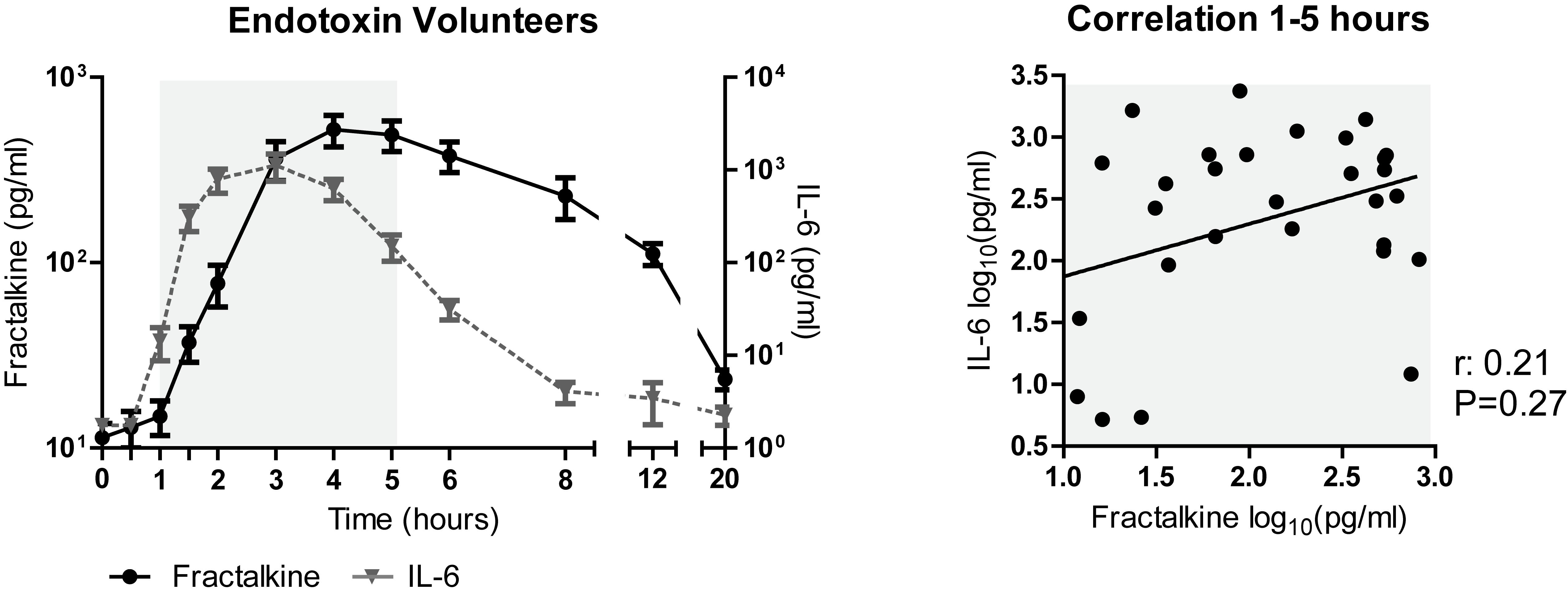
**

Supplement: Additional file 4: Figure S4. — Fractalkine compared to IL-6 release after intravenous injection of endotoxin to healthy humans in vivo. Fractalkine release after intravenous injection of endotoxin (lipopolysaccharide, 4 ng/kg body weight) into five healthy subjects compared with IL-6 release. Right panel, correlation between fractalkine levels and IL-6 measured during the first 5 hours after endotoxin administration (gray). Data are expressed as mean ± standard error of the mean. (DOC 135 kb) [file 13054_2015_1125_MOESM4_ESM.doc]
